# The effects of psychological treatment of perinatal depression: an overview

**DOI:** 10.1007/s00737-021-01159-8

**Published:** 2021-07-06

**Authors:** Pim Cuijpers, Eirini Karyotaki

**Affiliations:** 1 Department of Clinical, Neuro and Developmental Psychology, Amsterdam Public Health Research Institute, Vrije Universiteit Amsterdam, Van der Boechorststraat 7-9, 1081 BT Amsterdam, The Netherlands; 2 WHO Collaborating Centre for Research and Dissemination of Psychological Interventions, Vrije Universiteit Amsterdam, Amsterdam, the Netherlands; 3 Faculty of Medicine, University of Turku, Turku, Finland

**Keywords:** Perinatal depression, Major depression, Psychotherapy, Cognitive behavior therapy, Interpersonal psychotherapy, Meta-analysis

## Abstract

Perinatal depression is an important public health problem. Psychological interventions play an essential role in the treatment of depression. In the current paper, we will present the results of a series of meta-analyses on psychological treatments of perinatal depression. We report the results of a series of meta-analyses on psychological treatments of depression, including perinatal depression. The meta-analyses are based on a database of randomized trials on psychotherapies for depression that has been systematically developed and updated every year. Psychological interventions are effective in the treatment of perinatal depression with a moderate effect size of g = 0.67, corresponding with a NNT of about 4. These effects were still significant at 12 months after the start of the treatment. These interventions also have significant effects on social support, anxiety, functional impairment, parental stress, and marital stress. Possibly the effects are overestimated because of the use of waiting list control groups, the low quality of the majority of trials and publication bias. Research on psychotherapies for depression in general has shown that there are no significant differences between the major types of therapy, except for non-directive counseling that may have somewhat smaller effects. CBT can also be delivered in individual, group, telephone, and guided self-help format. Interventions in subthreshold depression are also effective and may prevent the onset of a full-blown depressive disorder, while therapies may be less effective in chronic depression. Psychological interventions are effective and deserve their place as first-line treatment of perinatal depression.

## Introduction

Depression during pregnancy and after the birth of an infant is highly prevalent and is associated with a considerable reduction in quality of life, social functioning (Drury et al. 2016), and also with parental and maternal functioning (O'Hara and McCabe 2013; Bernard et al. 2018; Cuijpers et al. 2021a). Perinatal depression is also associated with negative outcomes in infants and children, including diminished cognitive, emotional, behavioral, and physical outcomes (Stein et al. 2014; O'Hara and McCabe 2013; Liu et al. 2017). Previous research has shown that 9% of women in high-income countries and 17% of women in low- and middle-income countries are affected by perinatal depression according to a diagnostic interview (Woody et al. 2017). Treatment of perinatal depression is, therefore, critical to reduce suffering in affected women, their partners, and their children and to reduce the disease burden on the population level.

Psychological interventions play an important role in the treatment of perinatal depression. They have been recommended as the first-line treatment for perinatal women with a new depression episode (O’Connor et al. 2016). Furthermore, most women prefer psychotherapy over pharmacotherapy because of concerns about the effects of medication on the pregnancy (Dennis and Chung-Lee 2006).

Several dozens of randomized controlled trials have examined the effects of psychological treatments of perinatal depression. In the current paper, we summarize the results of our recent meta-analysis on these studies (Cuijpers et al. 2021a, b). Moreover, in our previous work, we found that the effects of psychological treatments of perinatal depression do not considerably differ from treatments in other populations (e.g., adults in general) (Cuijpers et al. 2018). This means that lessons learned from research on psychotherapies in general are also valid in the case of perinatal depression. Therefore, in the present paper, next to summarizing our perinatal depression meta-analysis findings, we also summarize the results of a series of meta-analyses of psychotherapies for adult depression in general conducted by our group over the past 15 years (Cuijpers 2017; www.metapsy.org). Also, we examine different types of psychotherapy, their format, number of sessions, subtypes of depression, longer-term effects, and effects on other outcomes than depression (e.g., parental and marital distress).

## Psychological treatment of perinatal depression

We recently conducted a meta-analysis of all randomized controlled trials on psychological treatments of perinatal depression based on an existing database of trials on psychotherapies for depression in general (Cuijpers et al. 2021a, b). The database is updated every year for new studies using systematic searches in Pubmed, PsycINFO, Embase, and the Cochrane Library. All studies comparing psychological treatments for any target group with a control condition, with another psychological treatment, with pharmacotehrapy or combined treatment are included. Effect sizes are calculated as the difference between the treatment and control condition divided by the pooled standard deviation (Hedges’ g), and a random effect model is used to pool the outcomes. In this paper, we will use Hedges’ g as indicator for outcome. A value of 0.2 can be considered as small, 0.5 as moderate, and 0.8 as large (Cohen 1988).

For the meta-analysis on psychological treatment of perinatal depression, we included 43 trials (6270 participants) with 49 comparisons between an intervention and a control group (e.g., treatment as usual). Psychological treatment was defined according to Campbell et al. (2013): “Psychotherapy is the informed and intentional application of clinical methods and interpersonal stances derived from established psychological principles for the purpose of assisting people to modify their behaviors, cognitions, emotions, and/or other personal characteristics in directions that the participants deem desirable”.

Eighteen studies were aimed at pregnant women, 24 at women with postpartum depression (one at a mixed population). In 22 studies, women had to meet diagnostic criteria for a depressive disorder to participate (the others used a cut-off on a self-rating depression measure). In the 43 studies, 49 psychological interventions were compared with a control group. Twenty-four of these were cognitive behavior therapy, 7 were interpersonal psychotherapy, 7 were supportive counseling, and 10 were other therapies. Twenty four of the 49 interventions used an individual format, 15 a group format, 3 Internet-based guided self-help, and 7 used a mixed or another format. Thirty-six interventions had between 6 and 12 sessions, 7 had fewer sessions, and 6 had more sessions (range 2 to 21). A total of 33 studies used a care-as-usual as control group, 5 used a waiting list, and the other 5 studies used another control group. Thirteen studies were conducted in North America, 10 in Europe, 6 in Australia, 6 in East Asia, and 8 in other countries. The risk of bias according to the Cochrane Risk of Bias tool was considerable in many studies. Fourteen of the 43 studies (33%) met all 4 quality criteria, 15 studies (35%) met two or three of the criteria, and the 14 remaining studies met no or only one criterion (33%).

The overall effect size was g = 0.67 (95% CI: 0.45 ~ 0.89) with high heterogeneity (*I*^*2*^ = 80%; 95% CI: 75 ~ 85). This effect size corresponds with a number needed to treat (NNT) of 4 which means that 4 women with perinatal depression have to be treated in order to find one more positive outcome compared to no treatment. This is a moderately large effect.

We conducted a series of sensitivity analyses, including analyses in which we only included studies with low risk of bias, analyses in which we excluded outliers, and analyses in which we adjusted for publication bias. In most of these analyses, the effects were comparable to those of the main analyses, although we did find some indications for publication bias. After adjustment for publication bias, the effect size dropped somewhat (g = 0.53), but it was still significant. Exclusion of outliers also resulted in a smaller effect size (g = 0.56); however, limiting the studies to those with low risk of bias did not (g = 0.87).

Fourteen studies also reported outcomes at 12 months after randomization. The effect sizes remained significant and moderate at follow-up (g = 0.40), and it remained comparable in the sensitivity analyses described above. Only four studies reported outcomes between 12- and 24-month follow-up, and the effects were no longer significant at that time point, possibly because of low statistical power (g = 0.27).

In our meta-analysis, we also examined other outcomes of psychotherapies for depression. We found that these therapies also had significant effects on social support, anxiety, functional impairment, parental stress, and marital stress. We found no effects on weight and height of the infants, although perinatal depression has been found in some studies to be associated with reduced weight and height of the infants (Slomian et al. 2021). The fact that we did not find effects on weight and height could be related to the limited number of studies and low statistical power. All results should be considered with caution because of the high levels of heterogeneity in most analyses.

## What do we know about the effects of psychotherapies for depression in general?

In a recent meta-analysis, we examined in a large sample of trials (256) whether the effects of therapies for depression for unselected adults differ when they are applied in specific target groups, like women with perinatal depression, older adults, patients with comorbid general medical disorders, primary care patients, student populations, and minorities (Cuijpers et al. 2018). In subgroup analyses, we found that the effects of psychotherapies in perinatal depression were somewhat smaller than in the other studies. However, in a multivariable meta-regression analysis in which we adjusted for the most relevant characteristics of the studies (such as proportion women, age group, ethnicity, several clinical characteristics, setting, risk of bias, type of control group, and treatment characteristics), we could not confirm that studies in perinatal depression differed significantly from studies in other adults. We can, therefore, cautiously conclude that knowledge about psychotherapies for depression in general is also valid in therapies for women with perinatal depression. This should be interpreted cautiously, however, because it is possible that some characteristics of studies may have an impact on the outcome in perinatal depression, but not in other target groups, or the other way around.

One important lesson learned from meta-analyses in psychotherapies in adults is that there are no significant differences between the main types of psychotherapy (Cuijpers et al. 2021a, b). In a large network meta-analysis, we included studies on eight types of psychotherapy that were examined in at least ten randomized trials: cognitive behavior therapy (CBT), behavioral activation therapy, problem-solving therapy, third-wave therapies (including acceptance and commitment therapy, mindfulness-based CBT), psychodynamic therapy, interpersonal psychotherapy, non-directive supportive counseling, and life review therapy (life review has only been examined in older adults). We included 331 trials on 34,285 patients that compared these 8 therapies with control groups (i.e., waitlist, usual care or pill placebo) or with each other. CBT was by far the most prevalent therapy type, with almost two-thirds of the trials including a CBT arm in our network meta-analysis (211/331 trials). Nevertheless, other types of therapy were also examined in 13 (life review) to 42 (non-directive counseling) trials. All therapies were more effective than control conditions, and there was no significant different difference between therapy types. Only non-directive supportive counseling had a significantly smaller effects compared to other therapy types, but this may be an artifact because this therapy was often used as a control condition and it was not clear whether it was delivered adequately in all studies.

In another network meta-analysis of 155 randomized trials (15,191 participants) on CBT for depression, we examined the different treatment formats (Cuijpers et al. 2019b). We included trials on individual, group, telephone-based, guided self-help (including Internet-based) therapies, and unguided self-help therapies. In these trials, treatments formats were compared either against each other or against a control condition (i.e., waiting list, usual care, or pill placebo control groups). We found that the effects of individual, group, telephone-based, and guided self-help did not differ significantly from each other. The effect sizes (Hedges’ g) ranged from 0.87 to 1.02 when compared to waiting list and 0.47 to 0.72 when compared to usual care. We did find that unguided self-help was significantly less effective than the formats in which human support was given. We also found that study dropout was larger in guided self-help compared to other treatment formats. This suggests that when guided self-help is delivered, clinicians should be careful of the increased risk of dropout.

In our meta-analysis of psychotherapies for perinatal depression (Cuijpers et al. 2021a, b), we also included three trials on Internet-based therapies, and these also had significant effects on depression (g = 0.89; 95% CI: 0.71 ~ 1.07). This means that Internet-based therapies are probably also effective in perinatal depression. Unguided interventions may also be effective, but their effects are probably smaller than those of guided interventions.

We recently conducted an “individual patient data” network meta-analysis in which we included the primary data from 39 RCTs (9751 participants) on guided and unguided interventions for depression (Karyotaki et al. 2021) in which perinatal depression studies were also included. We found that in general, guided and unguided Internet interventions have comparable effects in milder forms of depression, while guided interventions are more effective in more severe depression. We developed a web-based tool, where characteristics of an individual patient can be filled in, and then, a personalized prediction is generated about how large the effects are for guided and unguided Internet intervention (https://bit.ly/3b5gT77). In light of this evidence, unguided forms of Internet-based interventions may also be an option for mild symptoms of perinatal depression.

In another meta-analysis, we examined whether the amount, frequency, and intensity of therapy were related to the effect sizes (in this meta-analysis, we only included studies on individual therapies; Cuijpers et al. 2013). We found only a small association between number of therapy sessions and effect size, which was no longer significant when we adjusted for other characteristics of the studies in a meta-regression analysis. We did not find a significant association with the total contact time or duration of the therapy either. In this meta-analysis, we did find a significant association between the number of sessions per week and effect size. An increase from one to two sessions per week boosted the effect size by g = 0.45, while keeping the total number of treatment sessions constant. We recently conducted a randomized trial to verify whether frequency is indeed associated with the effect size, and we could indeed confirm that this is the case (Bruijniks et al. 2020). So, it can be recommended to have two sessions per week instead of one.

## Characteristics of patients and the association with outcome of psychotherapy

Several meta-analyses have focused on clinical characteristics of depression and characteristics of depressed patients that are also relevant for perinatal depression. One clinical characteristic that we examined in a meta-analysis is subthreshold depression (depressive symptoms but not meeting criteria for a depressive disorder; Cuijpers et al. 2014a). In this meta-analysis, we included 18 randomized controlled trials of different kinds of psychotherapy. The overall pooled effect size was g = 0.35 (95% CI: 0.23 ~ 0.47), which corresponds with a NNT of 5. This effect was, however, significantly smaller than the effects of therapy in patients with a major depressive disorder. This is not surprising in itself, because the level of depressive symptoms is low from the start in subthreshold depression, and thus, there is less room for improvement. In this meta-analysis, we also found that psychotherapies aimed at subthreshold depression have a significant effect on the incidence of major depressive episodes at 6-month follow-up (with a relative risk of 0.61) and possibly at 12 months (RR = 0.74).

This is also in line with another meta-analysis in which we examined the effects of interventions aimed at preventing the onset of depressive disorders in people who do not have a disorder at baseline (Cuijpers et al. 2020b). In this meta-analysis, we found that preventive interventions can reduced the incidence of depressive disorders at follow with a relative risk (RR) of 0.81 (95% CI: 0.72–0.91), indicating that those who had received the intervention had 19% less chance to develop a depressive disorder. There was a subset of 9 studies in perinatal depression, which had a comparable reduced risk (RR = 0.73; 95% CI: 0.52 ~ 1.00), although this was not significant, possibly because of limited statistical power.

Although chronic depression is a severe condition, relatively few studies have focused on the effects of psychotherapies for these patients. In a meta-analysis of 16 trials, we found that psychotherapy had a small but significant effect (g = 0.23; 95% CI: 0.06 ~ 0.41) on depression when compared with control groups (Cuijpers et al. 2010). However, psychotherapy was significantly less effective than pharmacotherapy in direct comparisons (g =  − 0.31), especially SSRIs. This finding, however, was fully attributable to dysthymic patients (the studies examining dysthymia patients were the same studies that examined SSRIs). Combined treatment was more effective than pharmacotherapy alone, but even more so with respect to psychotherapy alone, although again this difference may have reflected the greater proportion of dysthymic samples in the latter.

In several other studies, we examined whether baseline severity is associated with the outcomes of psychotherapies. In one study, we examined the association between baseline severity and outcome in a meta-regression analysis but could not find a significant association (Driessen et al. 2010). However, these results should be considered with caution, because severity was only measured on the study and not the individual-patient level. However, in an “individual participant data” (IPD) meta-analysis comparing CBT with pill placebo, we did not find an association between baseline severity and outcome either (Furukawa et al. 2017). In one more IPD meta-analysis, we also found no indication that baseline severity was associated with differential effects of CBT and antidepressants (Weitz et al. 2015).

There is some research on the question whether the effects of psychotherapies differ between men and women. Conventional meta-analyses are not well suited to examine this question because they can only examine the association between outcome and proportion of men or women at the study level, not at the individual level. IPD meta-analyses do allow to examine the association between outcome and gender at the individual level. None of the IPD meta-analyses we conducted suggested that there are differential effects between men and women (e.g., Cuijpers et al. 2014a, b; Karyotaki et al. 2017; 2018).

## The effects of psychotherapies are overestimated

One problem of meta-analytic research in psychotherapies for depression is that the effects are considerably overestimated if one simply looks at the overall pooled effect size (Cuijpers et al. 2019a, b). One reason is that many trials use waiting list control groups, which probably overestimate the effects when compared to other control conditions such as usual care or pill placebo. Another problem is that the quality of the majority of randomized trials is suboptimal and most trials (71%) have at least some risk of bias (Cuijpers et al. 2019a, b). Furthermore, publication bias is a major problem affecting the overall pooled effect sizes in meta-analyses of psychotherapies for depression (Driessen et al. 2015; Cuijpers et al. 2019a).

In one meta-analysis, we found an overall effect for all 325 comparisons between psychotherapies and control conditions of g = 0.63. However, after excluding studies using waiting list control groups, excluding studies with some risk of bias and after adjustment for publication bias, the resulting effect size was g = 0.31, less than half of the overall effect size. Most of the studies were aimed at CBT (165 comparisons), and the overall effect size of CBT was 0.62. After adjustment for the described problems, the effect size of CBT was reduced to g = 0.29. These effect sizes are still clinically relevant and are comparable to those of antidepressive medication (Cuijpers et al. 2019a). Direct comparison between psychotherapy and antidepressive medication also indicate that these two treatments have comparable effects (Cuijpers et al. 2020a).

In our meta-analysis of psychotherapies for perinatal depression (Cuijpers et al. 2021a, b), we did not find that studies with low risk of bias had a lower effect size. We did find that the effect size was larger in studies with waiting list control groups, and we also found indications for publication bias. It is, therefore, probable that the effect sizes of psychotherapy for perinatal depression have also been overestimated because of the described methodological problems.

## Conclusions

We found that psychological interventions are effective in the treatment of perinatal depression with moderate effect sizes and a NNT of about 4. We also found that these effects were still significant at 12-month follow-up. These interventions also have significant effects on social support, anxiety, functional impairment, parental stress, and marital stress. These effects may have been overestimated because of the use of waiting list control groups, the low quality of the majority of trials, and possible publication bias. However, after adjustment for these problems, the effect of these interventions was still significant.

There is a large body of research on psychotherapies for depression in general, not limited to perinatal depression, and there is no good reason to assume that this knowledge will not be valid in perinatal depression. This broader field has shown that there are no significant differences between the major types of therapy, except non-directive counseling that may have somewhat smaller effects. It has been demonstrated that CBT can also be effectively delivered in individual, group, telephone, guided self-help, and unguided self-help. Nevertheless, the effects of unguided interventions are somewhat smaller compared to the other formats, but they are still promising for mild depressive symptoms. Further, interventions in subthreshold depression are also effective and may prevent the onset of depressive disorders. Finally, there is no indication that therapies are less effective in severe depression and, for example, in ethnic minorities. However, psychotherapy may be less effective in chronic depression.

This study has several limitations that have to be taken into account when interpreting the findings. First of all, the number of studies in perinatal depression was relatively small, and the number of studies with low risk of bias was even smaller. Furthermore, it is not completely certain that the findings in the broader field of psychotherapy for depression are also valid in perinatal depression. Probably, these findings can be extrapolated to the perinatal field, but there is no guarantee that this is indeed the case. More research is needed to confirm this. Another problem is the high heterogeneity in this research field, and this heterogeneity cannot be completely explained by characteristics of the studies.

Despite these limitations, we can conclude, however, that psychological interventions are effective and deserve their place as first-line treatment of perinatal depression.
